# Delivery Mode Impacts Gut Bacteriophage Colonization During Infancy

**DOI:** 10.1080/29933935.2025.2464631

**Published:** 2025-03-14

**Authors:** Poorani Subramanian, Hector N. Romero-Soto, David B. Stern, George L. Maxwell, Shira Levy, Suchitra K. Hourigan

**Affiliations:** a National Institute of Allergy and Infectious Diseases, National Institutes of Health, Bethesda, Maryland, USA; b Women’s Service Line, Inova Health System, Falls Church, Virginia, USA

**Keywords:** Bacteriophages, virome, infant, delivery mode, cesarean section, vaginal delivery, microbiome

## Abstract

Cesarean section delivery is associated with altered early-life bacterial colonization and later adverse inflammatory outcomes. Although gut bacteriophages can alter the gut microbiome and host responses, little is known about how delivery mode impacts bacteriophage colonization over time. Therefore, we conducted shotgun metagenomic sequencing on serial stool samples from infants from birth to 24 months of age. Sixty percent of infants were born by vaginal delivery. Ninety-four percent of the DNA viral sequences identified were bacteriophages. Virome alpha diversity was increased in vaginally delivered infants at 2 months (*p* = 0.004). Beta diversity differed by delivery mode up to 12 months when stratified by peripartum antibiotic use (*p* < 0.05). Predicted bacteriophage hosts differed by delivery mode (Q < 0.1) up to 24 months. Moreover, predicted bacteriophage functional genes differed by delivery mode up to 24 months. There was a higher abundance of viral auxiliary metabolic genes associated with host responses in vaginal delivery at early timepoints. Clear differences in bacteriophage composition and function by delivery mode were seen in early life. Given that bacteriophages are known to affect immune responses, our results suggest that future investigation into how delivery mode leads to adverse inflammatory outcomes should also include the potential role of bacteriophages and transkingdom interactions.

## Background

Infancy represents a key window for gut microbiome establishment and plays a critical role in immune and metabolic programming.^6,16,20^ Disturbing the timing and order of microbial colonization can have lasting immune and inflammatory consequences.^13,40^ Cesarean section (CS) delivery represents an important factor perturbing the bacterial colonization of an infant and has been associated with later risk of several inflammatory conditions.^5,21,39^ We read with interest the recent work of Shah *et al*, revealing previously unappreciated virome diversity in the infant gut microbiome at 1 year of age, and uncovering unknown viral species and virus family-level clades.^38^ Although delivery mode is known to impact gut bacterial colonization, there is limited work investigating how delivery mode affects gut bacteriophage colonization, which is important given that gut bacteriophages can alter microbiome composition and function and directly affect host immune responses.^11,14,15,34^ Transmission of the gut virome from mother to infant does occur at birth, although reports vary in the amount of virome transmitted.^27,42^ There is some sharing of maternal and infant viral communities through cohabitation, and virome colonization is also determined by dietary and environmental factors in addition to direct maternal acquisition.^27,42^ One report showed that in the first week of life, delivery mode did not determine how much of the virome was shared between mother and infant, but some effect of delivery mode on bacteria-bacteriophage interaction was seen.^27^ However, how delivery mode affects virome colonization longitudinally in infancy and early childhood has not been studied. Therefore, building on this previous work, we aimed to assess the impact of delivery mode on the infant gut virome over the first two years of life.

## Methods

### Subjects, sample, and clinical data collection

Mothers were enrolled prenatally with informed consent in an Institutional Review Board approved longitudinal, prospective cohort study “The First 1000 Days of Life and Beyond” (Inova protocol #15–1804, WCG protocol #20120204). Inclusion criteria were that the Mother was pregnant, in good general health, age 18 years or older and the biological parent of child; there were no exclusion criteria. Serial stool samples were collected on dry swabs from infants at 1–2 days after birth and at around 2, 6, 12, and 24 months of age as previously described.^26,31^ These timepoints were chosen for 2 reasons: 1) there is more dynamic development of the gut microbiome in the first year of life, and so more frequent sampling was chosen in the first year of life and 2) these timepoints also represent some of the timepoints when infants are seen for well child checks at their pediatrician offices and so additional information from medical records from these visits could be obtained. All samples except for the 1–2 day samples were collected at home and mailed back to the lab, using previously validated methods and stored at −80°C until analysis.^29^ A minimum of 75 mg of stool per sample was used for analysis. Subjects for this current study were chosen from the larger cohort if they had adequate stool samples available at all time points. Demographic information, pregnancy details including mode of delivery mode and maternal antibiotic use, and infant data including feeding mode and antibiotic use were collected through a questionnaire, review of electronic medical records and serial surveys. Association between clinical and demographic factors with delivery mode was evaluated using the Chi-square test and two-sample t-test for categorical and continuous variables respectively.

### DNA extraction and Shotgun metagenomic sequencing

DNA was extracted from stool using the DNeasy PowerSoil Pro kit (Qiagen, Valencia, CA) following manufacturer’s instructions. Extractions were performed in single tubes on QIAcube robots (Qiagen) to reduce errors and sample-to-sample cross contamination and to provide standardized sample treatment. After extraction, DNA was quantified using the Quant-iT™ PicoGreen ™ Assay Kit (Thermo Fisher Scientific). Shotgun libraries were generated from 0.4 ng/ul DNA using the NexteraXT Library Prep kit and IDT for Illumina unique dual indexes at 1:4 scale reaction volume. After library preparation, libraries were cleaned using the SPRI beads in 50% PEG 8000 solution and their success was assessed by the Quant-iT™ PicoGreen™ dsDNA assay. Samples with library yields <1 ng/ul were re-prepped as needed. An equal volume of library from all plates was pooled and sequenced using a 300 cycle Nano kit on the Illumina MiSeq. Libraries were re-pooled based on the demultiplexing statistics from the MiSeq Nano run. Final pooled libraries were assayed on the Agilent BioAnalyzer to check the size distribution and absence of additional adaptor fragments. Libraries were sequenced on an Illumina NovaSeq 6000 S2 v1.5 flow cell, producing 2 × 150 bp paired-end reads.

Extraction blanks and nucleic acid-free water were processed and sequenced in exactly the same way along with experimental samples to empirically assess environmental and reagent contamination. Less than 100 reads were seen in these negative controls indicating a low risk of contamination across the sequencing run. An in-house laboratory-generated mock community consisting of DNA from *Vibrio campbellii* and Lambda phage was included as a positive sequencing control.

### Virome and bacteriome analysis

Read pairs were trimmed at Q15 with BBDuk v38.0.1 as well as for adapters using BBDuk’s collection of Illumina Nextera adapters. Trimmed reads were classified with Kraken v2.1.2 using their standard database, and any reads matching human were removed.^44^ The remaining reads were aligned to the human genome assembly GRCh38 using Bowtie2 v 2.3.4 with the parameter – very-sensitive. Any reads aligning to the human genome were removed, and the rest were assembled using metaSPAdes v3.5.15 using default parameters.^30^ Contigs longer than 1000 bp were then processed following a standardized protocol using VirSorter2 v2.2.3, CheckV v0.8.1, and DRAM-v v1.4.6 to identify viral sequences, check for completeness and annotate.^17,32,33,37^ Briefly, VirSorter2 was run including groups dsDNAphage, ssDNA with minimum length 5000 and minimum score 0.5; CheckV was run with default parameters; and DRAM-v with minimum contig size 1000. Additionally, contigs that were identified as putatively viral were aligned against the Gut Phage Database^9^ and further filtered by running geNomad v1.7.6 with default parameters.^8^ Gene finding on the matching contigs was done with Prodigal, and the resulting protein sequences and their respective contigs were clustered with VCONTACT2 using Diamond and the ClusterOne options.^3,19^ Cluster abundances were estimated with RSEM’s TMM method using reads aligned by Bowtie2 to the sequences identified as viral. These abundances were used to create a cluster abundance table.

Bacterial hosts of phage sequences were identified using iPHoP v1.3.3.^36^ Putative genes identified by DRAM-v were annotated by aligning to NCBI’s nr database using DIAMOND blastp with max-target-seqs = 2 and all other parameters default.^7^ Results were filtered for query coverage >85% and e-value <1e-5. Genes from the same contig which aligned to same NCBI phage genomes were assigned to that taxonomy. Any contigs which contained genes which aligned to different/conflicting genomes were not counted. DRAM-v was also used to identify viral auxiliary metabolic genes (vAMGs). Taxonomic classification of the whole microbiota, including the bacteriome, was done using Kraken V2.1.2 using a custom database made from the genomes in GTDB v80, EuPathDB48, and the human, viral and protist standard Kraken database. Rarefaction curves based on this classification were used to assess adequate sequencing depth, determining a threshold of 3 million reads to capture the diversity in each sample.

### Statistical methods

The Shannon alpha diversity index of the viral clusters was computed on the abundances using the diversity function from the vegan R package. Statistical comparison of the diversity by delivery mode within each time point was done with the non-parametric Kruskal–Wallis test using the kruskal.test R function. To assess if diversity was increasing over time and if change differed by delivery mode, the lmer function from the lmerTest R package was used with the model shannon ~ time * delivery_mode + (1|SubjectID). Beta diversity was compared between groups using the Bray-Curtis dissimilarity measure and PERMANOVA was performed with the adonis2 function from the vegan R package. All other statistics were done with linear mixed effects models using the lmerTest R package on its own and through the MaAsLin2 R package v1.15.1 with log transformation to assess differential abundances of virome features.

## Results

Shotgun metagenomic sequencing was conducted on 272 serial stool samples from 55 infants, collected at around 1–2 days of life and 2,6,12, and 24 months. 33/55 (60%) infants were born by vaginal delivery (VD). See Supplementary Table S1 for the summary statistics of the virome sequencing and metagenomics assembly. CS-delivered infants were more likely to have exposure to maternal peripartum antibiotics (*p* < 0.001). There were no differences by delivery mode in sex, ethnicity, race, breast feeding and infant antibiotic use (Suplementary Table S2). Day of collection of stool samples were similar between those born by VD and CS (Suplementary Figure S1).

From the virome analysis of the sequence data, we identified DNA viruses and attempted to resolve taxonomies. A total of 5976 unique viral clusters were identified. Of these, only 97.9% could be identified by geNomad. Clusters from the Caudoviricetes class (bacteriophages) were the most common (95.9%). After aligning the putative gene sequences to NCBI’s nr database, we classified 94% of sequences on average for each time point from which we could infer the phages. The number of putative viral genes increased over time (*p* < 0.005, Supplementary Figure S2).

**Figure 1.**
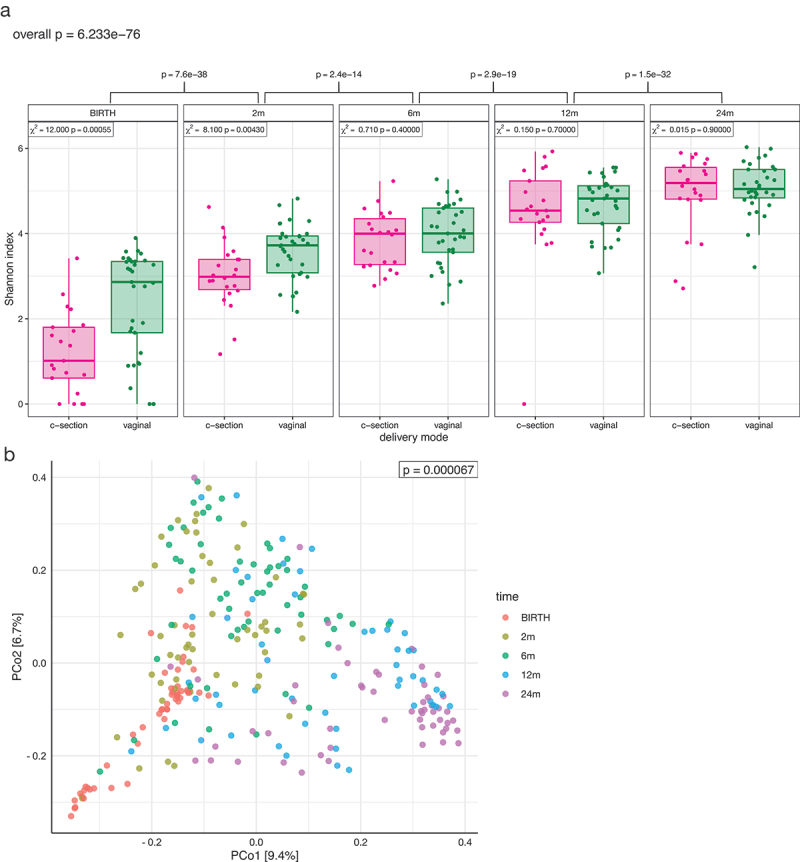
A: Alpha diversity (Shannon index) of the virome over time and by delivery mode. The p-value at each time point compares the diversity between delivery modes using the Kruskal–Wallis test. All other values are computed using the linear models and the lmerTest R package. The p-values in the brackets above the plots are from pairwise comparisons of diversity between adjacent timepoints (taking into account delivery mode using the LM ~time*delivery mode). The overall p-value is of the test indicating that the diversity does increase overall over time computed with LM ~time * delivery_mode + (1|SubjectID). B: Beta diversity (Bray–Curtis dissimilarity) of the virome over time with p-value computed using PERMANOVA test.

We then assessed the alpha and beta diversity of the virome by delivery mode and time. Alpha diversity (Shannon index) increased over time (*p* < 0.005, Figure 1A). There was increased alpha diversity in VD compared with CS at birth (*p* < 0.005) and 2 months (*p* = 0.004), with significance remaining after adjusting for peripartum antibiotic use. No difference in alpha diversity by delivery mode was seen at later timepoints. The effect size of delivery mode on alpha diversity appeared to be higher in the virome than the bacteriome at early time points (Suplementary Table S3). No differences were seen in alpha diversity by other clinical factors, including breast feeding and infant antibiotic use, although others have reported that breast feeding can alter infant virome colonization.^22^ Beta diversity (Bray-Curtis) differed by timepoint (*p* < 0.005, Figure 1B) but not overall by delivery mode. However, within each time point, beta diversity was significantly different by delivery mode when stratified by peripartum antibiotic use at 2 (*p* = 0.042), 6 (*p* = 0.016), and 12 months (*p* = 0.019) (Supplementary Figure S3).

**Figure 2.**
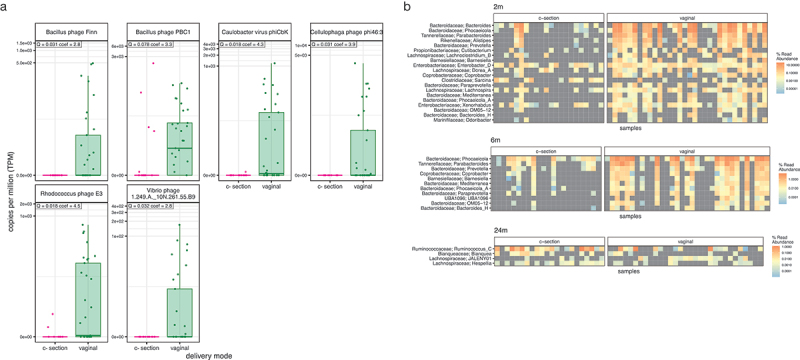
A: Significantly differentially abundant bacteriophages by delivery mode at 2 months. The y-axis is the abundance in copies per million which is computed by dividing the number of reads mapping to the genome by the length of the genome times 1 million. B: Significantly differentially abundant predicted bacterial hosts over time and by delivery mode.

**Figure 3.**
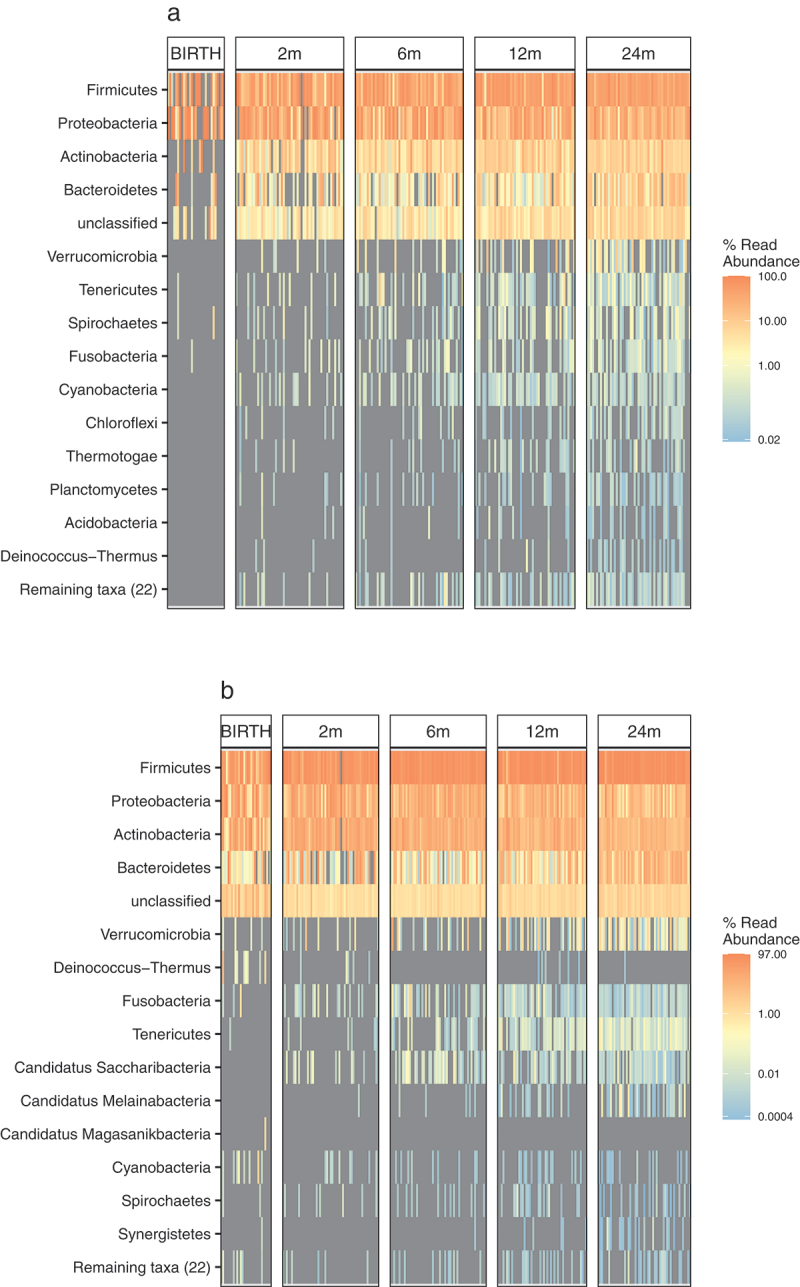
A: Predicted host bacterial abundance and B: whole microbiome bacterial abundance at a phylum level. The unclassified taxa signify sequences that could not be classified by Kraken or iPhoP.

We also assessed the phage abundances and putative host bacterial taxa abundance over time and delivery mode. The vast majority of viral clusters were from bacteriophages (94%). Six identified phages were shown to significantly differ by delivery mode (FDR <0.1) at 2 months (Figure 2A), but not at later timepoints; all were higher in VD than CS. Certain predicted host bacterial species differed significantly in abundance by delivery mode at each time point after birth, with an increased number of significantly differentially abundant hosts identified in VD compared with CS up to 6 months (Figure 2B). Notably, phages belonging to the *Bacteroidaceae* hosts were increased in VD at 2 and 6 months. At 24 months, there were more differentially abundant phage hosts predicted in CS compared to VD. The predicted host bacterial taxa correlated closely with the bacterial abundance of the whole microbiome at all timepoints (Figure 3).

**Figure 4.**
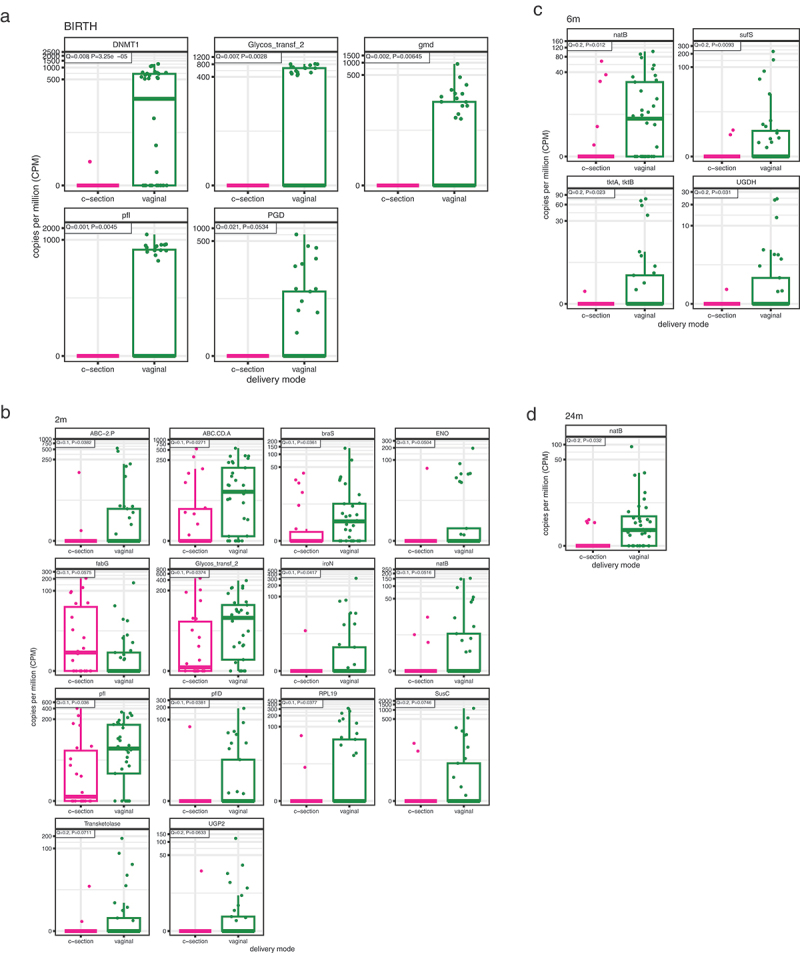
Differentially abundant viral auxiliary metabolic genes (vAMGs) by delivery mode and timepoint.

We then attempted to assess the predicted functional genes and pathways of the identified virome over time and by delivery mode. Very few functional pathways were identified and did not differ by delivery mode. However, when looking at vAMGs, differences were found by delivery mode at birth, 2, 6, and 24 months. Of the differentially abundant gene sequences, only fabG, was found to be in higher abundance in CS than VD at 2 m. All others were higher in abundance in VD compared to CS and included ENO, tkA, Glycos_transf_2, PGD, UGDH, ABC.CD.A and RPL19, genes associated with human host response. Others of note, essential to bacteria, included SusC, iroN, DNMT1 and UGP2. Full results are shown in Figure 4 and Supplementary Table 4.

## Discussion

Our study shows distinct differences in bacteriophage diversity, composition, function and predicted hosts by delivery mode over the first two years of life.

Alpha diversity was increased in VD compared with CS at birth and 2 months only, without differences being found at later time points. Interestingly, the only other study examining the virome by delivery mode later in infancy, in 20 infants at a single time point of 12 months, still found increased alpha diversity in those vaginally delivered at 1 year.^28^ In addition, regardless of delivery mode, we showed alpha diversity increased over time.^25^ This differs from some previous studies which report decreasing bacteriophage diversity over the first few years of life, possibly due to differences in preparation of the samples and updated bioinformatics tools and databases utilized in our study.^23,42^ However, other reports concur with our study and show an increasing diversity of bacteriophages in infancy, including a recent study by Lou et al following infants up to three years of age.^2,22,25^ In our study, the analysis at birth is somewhat limited as fewer CS samples could be used (*n* = 6) due to poor quality reads and/or no identifiable viral sequences. Mother-infant vertical transmission of the gut virome has been shown to varying degrees;^14^ our results suggest this may be further lowered by CS, possibly due to less overall exposure to the maternal perineal microbiome. Bacteriophage composition by delivery mode, when stratified by peripartum antibiotic use, also showed differences by delivery mode up to 12 months. This suggests that peripartum antibiotic exposure, which is higher in CS delivery, may also have an additional affect in bacteriophage colonization. Predicted bacteriophage hosts also differed by delivery mode at each time point after birth. *Bacteroidaceae* hosts were increased in VD at 2 and 6 months, with the bacterial taxa *Bacteroides* known to be increased in VD compared to CS infants in early life.^30^ Interestingly, it was recently shown that early phage colonizers that show persistence up to three years of age were mostly maternally transmitted and had *Bacteroides* hosts;^25^ it could be postulated that vaginal delivery increases the amount of maternally transmitted *Bacteroides* hosts and warrants further investigation.

Importantly, gut bacteriophages can alter microbiome composition and function and directly affect host immune responses.^14,15,18,34^ There was a higher abundance of genes associated with host responses in VD compared to CS, predominantly at 2 and 6 months. These included the genes ENO (receptor for human plasminogen that can promote recruitment of monocytes), tkA (required to make erythrose-4-phosphate, which is a precursor of aromatic amino acids and vitamins), PGD (important to produce NADPH), and UGDH (catalyzes the oxidation of UDP-glucose to UDP-glucuronate).^10,18,24,34,44,45,47^ Several genes essential to bacterial functions were also increased in VD compared to CS at early timepoints. These included SusC (essential for utilization of maltooligosaccharides and starch in *Bacteroides thetaiotaomicron*) and iroN (essential for iron uptake in bacterial systems).^12,35^ Only fabG, associated with sugar metabolism in pathogenic bacteria, was found to be in higher abundance in CS than VD at 2 m.^46^

The reasons for the difference in bacteriophage colonization and function by delivery mode up to 24 months are still yet to be determined. While differential acquisition of the maternal virome is possible, as infants delivered by CS bypass the birth canal and maternal vaginal and perineal microbiome exposure, studies suggest that maternal to infant transmission of the virome in early life is already very low.^27,42^ It has been suggested dietary factors such as breastfeeding contribute to early life virome colonization, however there were no differences in breastfeeding by delivery mode in our study (Suplementary Table 2). It is likely that the differential bacterial colonization known to occur by delivery mode also affects bacteriophage differences, as evidenced by predicted bacteriophage hosts closely resembling whole-microbiome bacterial composition (Supplementary Figure S2).^1,4,5,43^

Although to our knowledge, our study is the first to examine the impact of delivery mode on infant virome colonization over the first 2 years of life, there are several limitations. Only DNA viruses were examined and so how delivery mode may impact gut RNA viruses remains unknown. The collection method for stool samples has been validated for the assessment of bacterial communities, but not , specifically, the assessment of bacteriophages.^29^ Additionally, maternal vaginal and stool viromes were not assessed and so maternal to infant transmission by delivery mode cannot be assessed.

## Conclusions

Clear differences in bacteriophage composition and function were seen by delivery mode over the first two years of life. Given that phages are known to affect host immune response, our results suggest that future investigation into how delivery mode may lead to adverse inflammatory outcomes should not only include bacterial microbial colonization but also the potential role of bacteriophages and transkingdom interactions.

## Supplementary Material

Supp table 2.docx

Supp_fig_1.jpg

Supplementary Table 3.docx

Revised_Supp_Figure_3.jpg

Supplementary Table 4.docx

Revised_Supp_Fig_2.jpg

Supp Table 1.xlsx

## Data Availability

The data sets generated and/or analyzed in the current study are available in the NCBI SRA repository (https://www.ncbi.nlm.nih.gov/bioproject/PRJNA988496) under BioProject number: PRJNA988496. Results of this work were also published as a preprint.^41^

## Abbreviations

CS: Cesarean section
VD: Vaginal delivery
FDR: False discovery rate
vAMGs: Viral auxiliary metabolic genes
fabG: 3-oxoacyl-[acyl-carrier-protein] reductase FabG
ENO: Enolase
tkA: Transketolase
Glycos_transf_2: Glycosyl transferase family 2
PGD: 6-phosphogluconate dehydrogenase
UGDH: UDP-glucose 6-dehydrogenase
ABC.CD.A: Putative ABC transport system ATP-binding protein
RPL19: Large subunit ribosomal protein L19
SusC: TonB-dependent starch-binding outer membrane protein SusC
iroN: Iron complex outermembrane recepter protein
DNMT1: DNA (cytosine-5-)-methyltransferase
UGP2: UTP glucose-1-phosphate uridylyltransferase
