# Supplementary figures and images for: Delivery Mode Impacts Gut Bacteriophage Colonization During Infancy
Source: Gut Microbes Rep. 2025 Mar 14;2(1):2464631. doi: 10.1080/29933935.2025.2464631 (PMC12352455; doi:10.1080/29933935.2025.2464631)

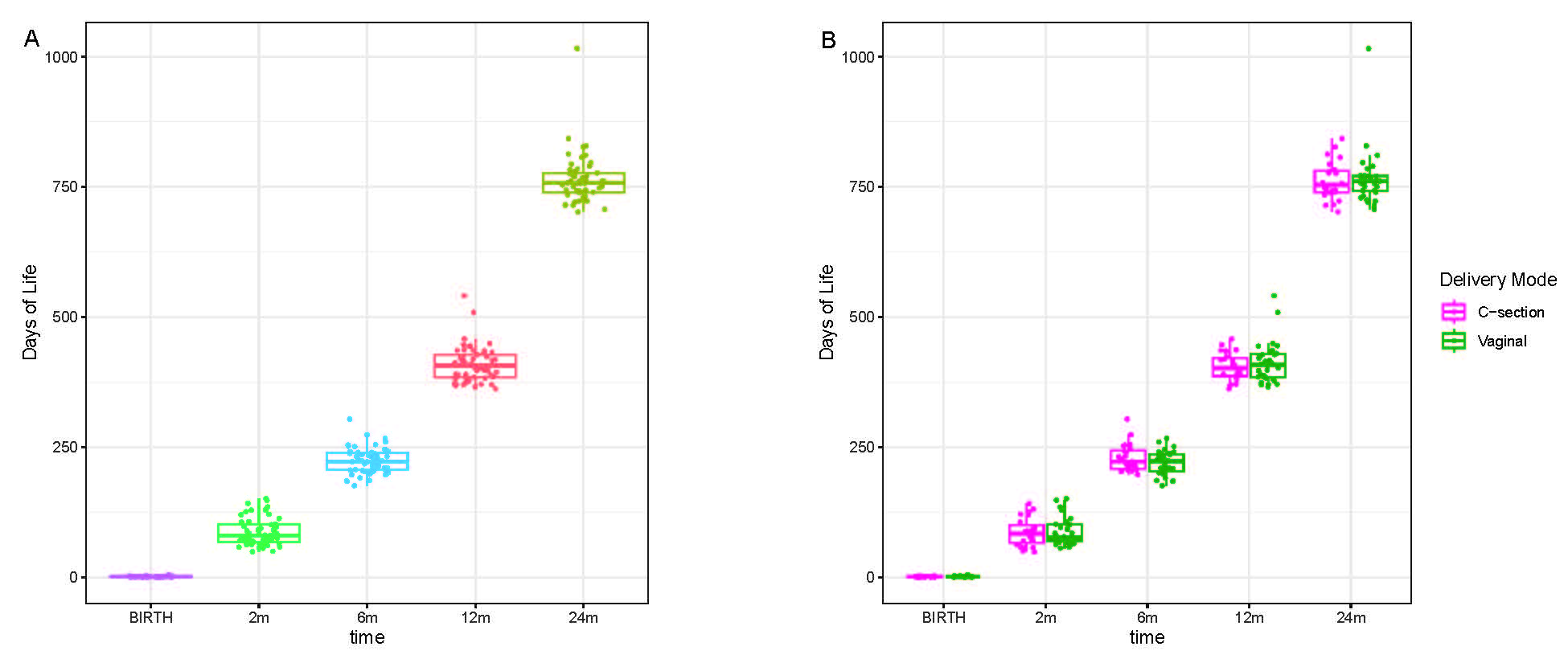

Supplement: Supp_fig_1.jpg [file KGMR_A_2464631_SM7352.jpg]

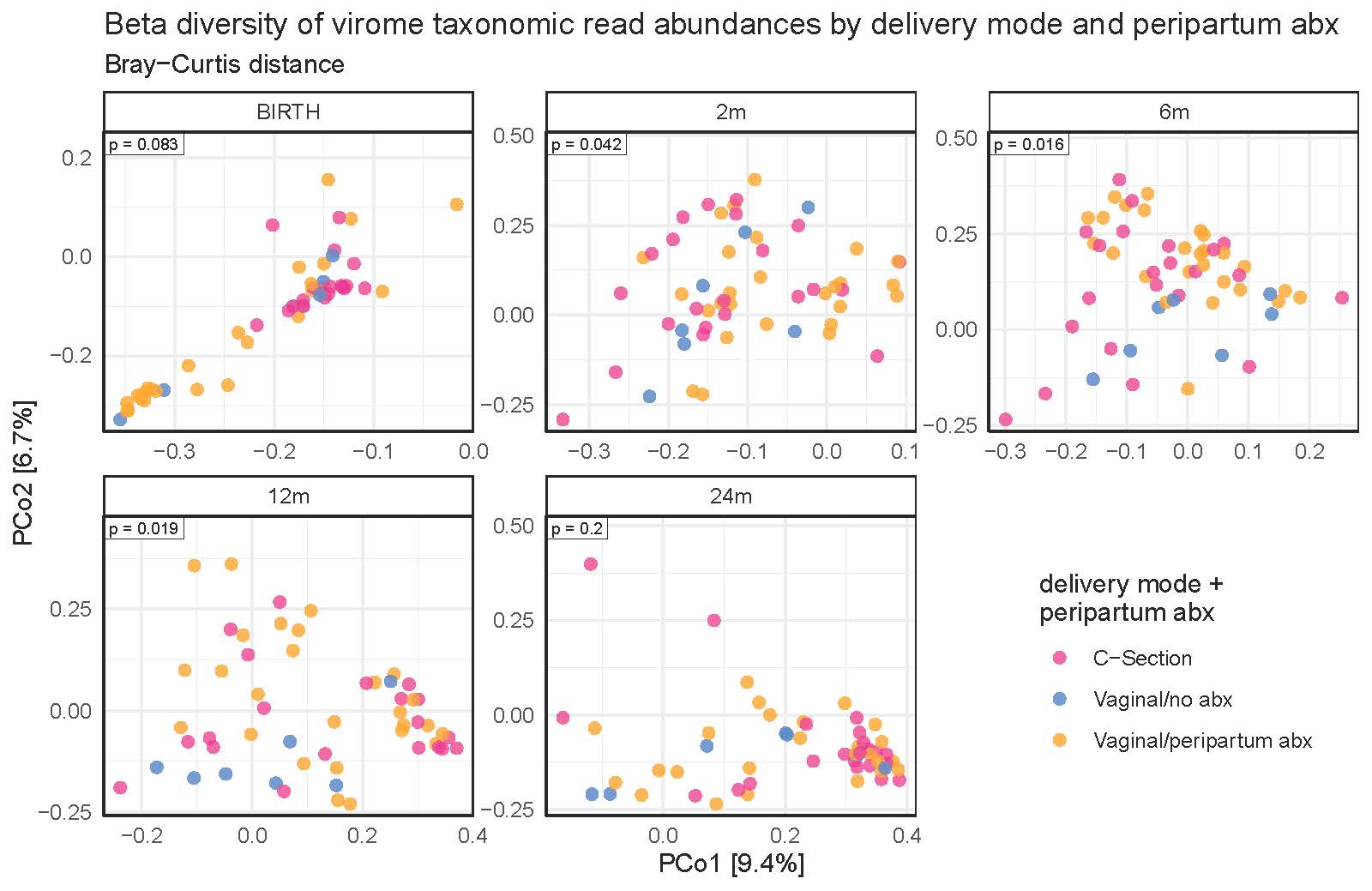

Supplement: Revised_Supp_Figure_3.jpg [file KGMR_A_2464631_SM7350.jpg]

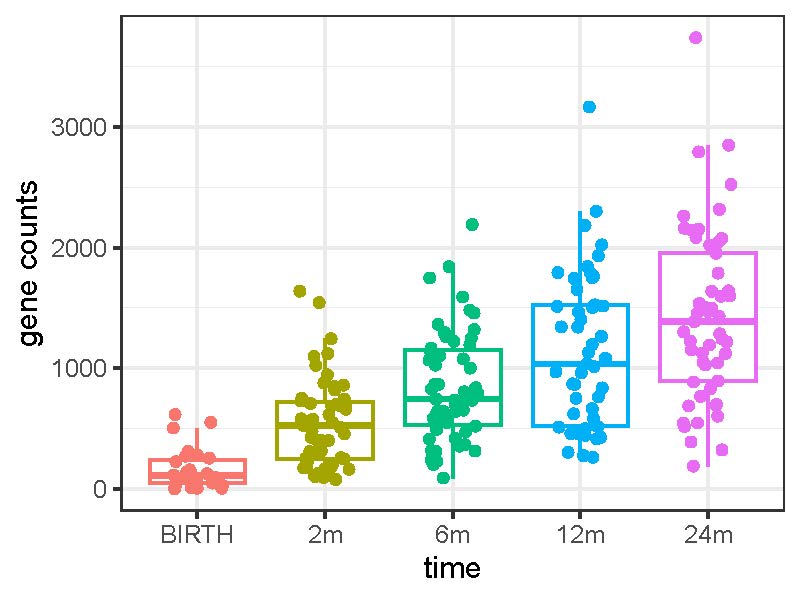

Supplement: Revised_Supp_Fig_2.jpg [file KGMR_A_2464631_SM7348.jpg]
