# Supplementary material for: Delivery Mode Impacts Gut Bacteriophage Colonization During Infancy
Source: Gut Microbes Rep. 2025 Mar 14;2(1):2464631. doi: 10.1080/29933935.2025.2464631 (PMC12352455; doi:10.1080/29933935.2025.2464631)
Supplement: Supplementary Table 3.docx [file KGMR_A_2464631_SM7351.docx]

| **Alpha diversity of virome by delivery mode** | | | | | | |  | | |
| --- | --- | --- | --- | --- | --- | --- | --- | --- | --- |
| time | p | | chisq | | effect_size | | | | |
| BIRTH | 0.000553 | | 11.92626 | | 0.225024 | |  | | |
| 2m | 0.004346 | | 8.133412 | | 0.150619 | |  | | |
| 6m | 0.399883 | | 0.708678 | | 0.013124 | |  | | |
| 12m | 0.702841 | | 0.145533 | | 0.002746 | |  | | |
| 24m | 0.901924 | | 0.015186 | | 0.000287 | |  | | |
|  |  | |  | |  | |  | | |
| **Alpha diversity of bacteriome by delivery mode** | | | | | | | | |  |
| time | | p | | chisq | | effect_size | | |  |
| BIRTH | | 0.004653 | | 8.009524 | | 0.151123 | |  |  |
| 2m | | 0.050166 | | 3.835891 | | 0.071035 | |  |  |
| 6m | | 0.203609 | | 1.616293 | | 0.029931 | |  |  |
| 12m | | 0.863593 | | 0.029516 | | 0.000547 | |  |  |
| 24m | | 0.134555 | | 2.239153 | | 0.042248 | |  |  |
